# Biological Activities of Polyphenols from Grapes

**DOI:** 10.3390/ijms11020622

**Published:** 2010-02-04

**Authors:** En-Qin Xia, Gui-Fang Deng, Ya-Jun Guo, Hua-Bin Li

**Affiliations:** Department of Nutrition, School of Public Health, Sun Yat-Sen University, Guangzhou 510080, China; E-Mails: enqinxia@163.com (E.X.); misyfly@163.com (G.D.); guoyajunleo@163.com (Y.G.)

**Keywords:** grape, polyphenol, bioactivity, antioxidant activity, cardioprotective action, anticancer activity, anti-inflammation activity, antimicrobial effect

## Abstract

The dietary consumption of grape and its products is associated with a lower incidence of degenerative diseases such as cardiovascular disease and certain types of cancers. Most recent interest has focused on the bioactive phenolic compounds in grape. Anthocyanins, flavanols, flavonols and resveratrol are the most important grape polyphenols because they possess many biological activities, such as antioxidant, cardioprotective, anticancer, anti-inflammation, antiaging and antimicrobial properties. This review summarizes current knowledge on the bioactivities of grape phenolics. The extraction, isolation and identification methods of polyphenols from grape as well as their bioavailability and potential toxicity also are included.

## 1. Introduction

Grapes have a long and abundant history. During the ancient Greek and Roman civilizations, grapes were revered for their use in winemaking. Nowadays, there are three main species of grapes: European grapes (*Vitis vinifera*), North American grapes (*Vitis labrusca* and *Vitis rotundifolia*) and French hybrids. Grapes are classified as table grapes, wine grapes (used in viniculture), raisin grapes, and so on, with edible seeds or seedless. People often enjoy the various grape products, such as fruit, raisins, juice and wine. Grape fruit contains various nutrient elements, such as vitamins, minerals, carbohydrates, edible fibers and phytochemicals. Polyphenols are the most important phytochemicals in grape because they possess many biological activities and health-promoting benefits [1–3]. The phenolic compounds mainly include anthocyanins, flavanols, flavonols, stilbenes (resveratrol) and phenolic acids [4–6]. Anthocyanins are pigments, and mainly exist in grape skins. Flavonoids are widely distributed in grapes, especially in seeds and stems, and principally contain (+)-catechins, (−)-epicatechin and procyanidin polymers. Anthocyanins are the main polyphenolics in red grapes, while flavan-3-ols are more abundant in white varieties [7–9].

From the clue of “French paradox”, polyphenolics from grapes and red wines attracted the attention of scientists to define their chemical composition and their properties for human health [10]. The reported evidences of beneficial health effects of phenolic compounds include inhibiting some degenerative diseases, such as cardiovascular diseases [11–14], and certain types of cancers [15–17], reducing plasma oxidation stress and slowing aging [18,19]. Phenolic compounds are also regarded as preservatives against microbes and oxidation for food [20,21]. What’s more, *in vivo* assays showed that phenolic compounds are bioavailable [10,22]. Therefore, besides wine and juice, grape diet supplements would be promising functional foods worthy of popularization. However, some reports have also shown that at higher concentrations the effect of phenolic compounds on health was negative and some structures in particular promoted the negative effects [23]. In addition, some high molecular weight phenolics could not be absorbed [24,25]. Apparently, research on direct ingestion of different doses and compositions of grape products are the urgent task in the field.

This review summarizes current knowledge on extraction, isolation and identification methods, bioactivities, bioavailability and potential toxicity of grape phenolics. Special attention is paid to the bioactivities, including antioxidant, cardioprotective, anticancer, anti-inflammation, antiaging and antimicrobial properties. Finally, this paper tries to show some directions for further research and applications of grapes.

## 2. The Distribution of Phenolic Compounds in Grape

Grape is a phenol-rich plant, and these phenolics are mainly distributed in the skin, stem, leaf and seed of grape, rather than their juicy middle sections (Table 1) [26,27]. Total concentration of phenolic compounds were about 2178.8, 374.6, 23.8, and 351.6 mg/g GAE (gallic acid equivalent) in seed, skin, flesh, and leaf, respectively [26]. The total phenolic content of grape skins varied with cultivar, soil composition, climate, geographic origin, and cultivation practices or exposure to diseases, such as fungal infections [28]. The compounds mainly included proanthocyanidins, anthocyanins, flavonols, flavanols, resveratrols and phenolic acids [4,5,29,30]. Proanthocyanidins are the major phenolic compounds in grape seed and skin of grape [30]. Anthocyanins are pigments and responsible for the color of grape fruits, and flesh did not contain anthocyanins [4,13]. In red wine, anthocyanins and flavonoids are the major two groups of phenolic compounds, and (+)-catechin is an abundant flavonoid [31].

## 3. Extraction, Purification and Identification of Phenolic Compounds from Grape

Liquid-liquid extraction is usually used for extraction of phenolic compounds from grapes. The extraction solvent is often ethanol, methanol, acetone or formic acid and water in different ratios. For grape skin, the crude extract mainly contained anthocyanins and flavonols. Grape seeds could be extracted by pressurizing and heating, and flavanols and hydroxycinnamic derivatives were obtained [38]. Although the solvent extraction offers high recovery of phenolic compounds from grapes, the use of large amounts of organic solvents poses health and safety risks to researchers, and is environmentally unfriendly. Thus, several improved methods have been developed to extract phenolics from grapes, such as microwave-assisted extraction [39], ultrasound-assisted extraction [5,40,41], supercritical fluid extraction [42,43], subcritical water extraction [44]. These extraction methods could significantly eliminate or reduce the use of organic solvents. In addition, a Lichroprep RP-18 column was employed to isolate catechin, oligomeric and polymeric procyanidin fractions from the crude extract of grape seeds using the distilled water adjusted to pH 7.0 to eliminate phenolic acids, followed by ethyl acetate to elute catechins and oligomeric fraction. The polymeric procyanidins absorbed at the top of the bed were eluted with methanol [45–47].

Total phenolic content was analyzed by a colorimetric assay using Folin–Ciocalteu’s phenol reagent [32]. Ferulic acid or gallic acid was used as standard, and the total phenolic content was expressed as mg/L of ferulic acid equivalent, or GAE against the fresh weight of the sample (mg/g) [48,49]. In the literature, much attention has been paid to the determination of anthocynins and flavonoids in grapes. The methods were mainly high-performance liquid chromatography (HPLC) with different detectors, in which HPLC-UV detection was a common tool [41,46,50], followed by HPLC-mass spectrometry (MS) detection [51]. Some complex devices have been employed by more than one MS. Before injection into the HPLC, the crude extract could be purified by solid-phase extraction (SPE) or improved liquid chromatography employed in order to obtain a more perfect profile of phenolic compounds in grape than ever possible before[4,6,53,54].

**Figure 1.**
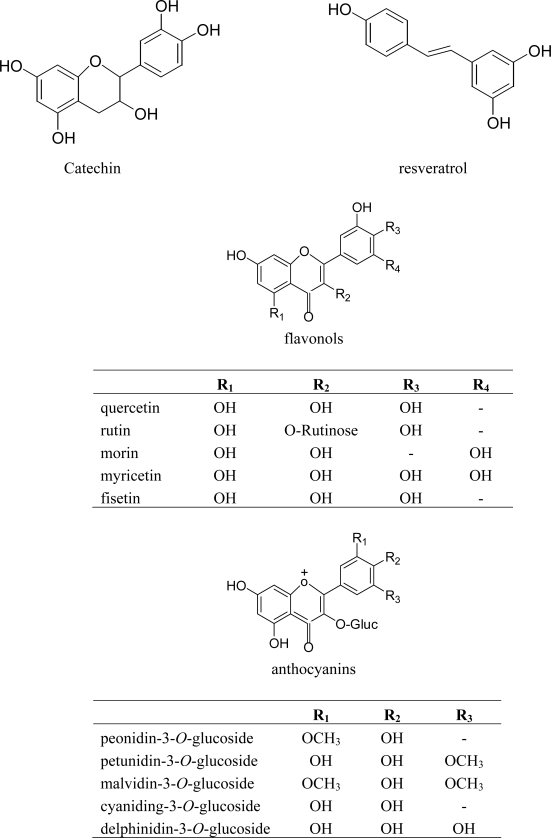
The chemical structures of some phenolic compounds from grapes.

In the literature, chemical structures of many phenolic compounds from grapes have been reported. The chemical structures of some important phenolic compounds are shown in Figure 1.

## 4. Bioactivity of Phenolic Compounds from Grape

Recently, growing interests on phenolic compounds from grapes have focused on their biological activities linking to human health benefits, such as antioxidant, cardioprotective, anticancer, anti-inflammation, antiaging and antimicrobial properties.

### 4.1. Antioxidant Activities

Being most the notable bioactivity of phenolic compounds from grapes, the antioxidative characteristics have been widely studied, including scavenging of free radicals, inhibition of lipid oxidation, reduction of hydroperoxide formation, and so on [18,19]. Several methods were employed to evaluate the antioxidant capacities of phenolic compounds extracted from various grapes or different parts of grapes, such as the 1,1-diphenyl-2-picryhidrazyl (DPPH) method [55], oxygen radical absorbance capacity (ORAC) assay [56], crocin bleaching assay (CBA) [57], 2,2′-azino-bis-(3-ethylbenzothiazoline-6-sulfonic acid) (ABTS) assay [58], the thiobarbituric acid reactant substances (TBARS) [59], Trolox equivalent antioxidant capacity (TEAC) assay [60], and the ferric reducing antioxidant power (FRAP) assay [61].

Using these methods above, notable antioxidant activities were found for grape wine and juice and the extracts from different parts of grapes. The values of antioxidant capacities were very different (Table 2). Seen from the Table 2, juice and wine, and even pomace from grapes had high antioxidant capacities. The extracts of defatted grape seeds expressed half less antioxidant capacity than that of whole grape seeds, which indicates that the process of oil extraction removed or damaged some antioxidant compounds. In different parts of grape, the highest antioxidant capacity was found in grape seeds, followed by skin, and the flesh displayed the lowest antioxidant capacity [26]. Therefore, the extracts from grape seeds are a promising antioxidant for dietary supplement.

The antioxidant activities of the extracts from grape and its products have been widely studied in different biological or food system. Seen from Table 3, the extracts from grape and its products could reduce oxidative stress of biological system and prevent food spoil.

Many researchers have tried to discover which phenolic compounds and chemical structure(s) are mainly responsible for the antioxidant activities of grape extracts. For same phenolic compounds, 50% and 25% (*v*/*v*) concentrations showed the same antioxidant activities, both being better than that of the 10% (*v*/*v*) concentration. The result suggested that perhaps the antioxidant capacity of phenolics has a concentration saturation limit, and above this limit, the activity could not increase further with the concentration [66]. However, the relationship between phenolic compounds and antioxidant capacity was inconsistent among the results from different studies, which indicated that, besides the concentration, the antioxidant capacities of phenolic compounds were affected by other factors [49,67]. In a study, malvidin-3-glucoside showed the highest antioxidant capacity in wine anthocyanins [35]. Although total phenolic index was lower in grape flesh than in grape skin because anthocyanins were absent in the flesh, they possessed equal amounts of reactivity to hydroxyl radicals [13]. In another study, the results also showed that the anti-radical activity was due to the flavanols, rather than anthocyanins [68].

The results showed that procyanidin polymers with higher degrees of polymerization had higher antioxidant activities [46]. However, Faria *et al*. [74] showed that in five fractions of different degrees of procyanidins polymers, the second degree fraction displayed the highest antioxidant capacity (scavenging peroxyl radicals). A similar result was obtained by Soobratteea *et al*. [75], who showed that the most antioxidative compound in various phenolics was procyanidin dimer, and the decrease in antioxidant capacity was in order of procyanidin dimer, flavanol, flavonol, hydroxycinnamic acids and simple phenolic acids. Diphenols are more effectively antioxidant than simpler phenols due to stabilization of the phenoxy-radical through hydrogen bonding [50]. The high molecule weight compounds might be as important as the monomer flavanols such as catechin, which have been demonstrated high antioxidant potential in phenolic compounds [76]. Furthermore, the antioxidant activity of a sample could be synergic effect among several compositions, rather than a single compound [47,77].

Pinelo *et al*. studied the impact of solvent on the antioxidant activity of catechin, resveratrol and grape extracts dissolved in ethanol, methanol and water. The maximum antiradical activity was in ethanol, then in methanol, and the minimum was in water [45]. By *in vitro* physiological procedure such as digestive enzymatic extraction, phenolic compounds from grape seed displayed a higher phenolic content and antioxidant capacity than by chemical procedure [78], which could be employed for the aim of getting dietary supplements from grapes.

The antioxidative characteristics of phenolic compounds are mainly ascribed to their free radical scavenging and metal chelating properties, as well as their effects on cell signaling pathways and on gene expression [75,79]. Arora *et al*. [80] found that flavonoids displayed higher antioxidant capacity against metal-ion-induced peroxidation than peroxyl-radical-induced peroxidation. The mechanism was mainly speculated to react directly to generate phenoxyl radicals [81], which was stable and cuts off the reaction chains. The chemical functional group and structure is OH for antioxidant capacity of phenolic compounds. The number of OH group and its position on the ring of molecule determined the antioxidant capacity of flavonols [80]. When the OH added onto the flavonoid nucleus, the activity enhanced, while substituted by the OCH_3_ groups, the activity diminished. The results were proved by Majo *et al*. [67,82]. The *o*-diphenoxyl groups in resveratol were determined to exhibit higher antioxidant activity than other compositions [83].

### 4.2. Cardioprotection Action

Postprandial hyperlipemia and oxidative stress, a well-defined risk factor for atherosclerosis, could be reduced by grape seed extracts or phenolic-rich grape juice. These oxidative stress factors refer to plasma lipid hydroperoxides, serum lipid peroxidation products, malondialdehyde-modified-LDL (MDA-LDL). The lipid-bound polyphenols increasing in serum were found even two hours after intake of phenolics, and MDA-LDL was detected after six weeks [48,69,84]. Grape seed extracts protected the rat liver against oxidative damage induced by irradiation *in vivo*, and remained the activities of superoxide dismutase and catalase at normal level [85].

Grape seed extracts (5–50 μg/mL) rich in polyphenols displayed reduction of platelet adhesion and aggregation and generation of superoxide anion, and were more effective than pure resveratrol [12]. Shanmuganayagam *et al*. [11] employed rabbits to investigate the potential of phenolic compounds to defend the hypercholesterolemic-induced platelet aggregation. After intake of the grape juice (225 mL/day), which was rich in polyphenolics, with hypercholesterolemic diet for 96 days, platelet aggregation in rabbits was significantly ameliorated and the development of atheroma was near 30% lower than that of the control group. Aortic fatty streak areas of hamster also showed significant reduction in the groups receiving catechin (84%) or quercetin (80%) or resveratrol (76%) in comparison to the controls [37,86]. Dell Agli *et al*. [79] showed anthocyanins from wine and grape skin inhibited phosphodiesterase-5 activity, which reduced the risk of cardiovascular diseases by vasorelaxation. Falchi *et al*. [13] made ischemic to isolated heart of rats for 30 min followed by two hours of reperfusion, and found that the ischemic reperfusion injury were significantly inhibited in the rats after 30 days consumption of the extracts of flesh and skin of grapes, and flesh and skin of grapes exhibited equal effect of cardioprotection.

Castilla *et al*. [87] found that phenolic compounds significantly ameliorated plasma lipid levels. After drinking 100 mL red grape juice/day for 14 days, the concentration of cholesterol-standardized-tocopherol and antioxidant capacity of plasma were significantly increased, and oxidized LDL and LDL were significantly reduced. The plasma level of HDL and apolipoprotein A-I were also elevated. In addition, consumption of red wine resulted to high concentrations of HDL cholesterol [14], which linked to control of the risk of coronary heart diseases. Ardevol *et al*. [88] reported that treatment of differentiated 3T3-L1 cells with procyanidin extracts reduced HSL in the mRNA levels, and inhibited triacylglycerol synthesis and boost its hydrolysis. After feeding to hamsters at a moderate dose of grape extracts, the plasma cholesterol was reduced 11% on average [86]. Moreover, plasma apolipoprotein A1 concentration was increased 26%, 22%, and 19%, induced by catechin, quercetin, and resveratrol, respectively [37].

For hemodialysis patients, phenolics of grapes are offered to prevent from inflammation. Red grape juice significantly reduced plasma monocyte chemoattractant protein 1, an inflammatory factor involved with cardiovascular disease risk, after three weeks’ consumption [87]. Tsang *et al*. [14] showed that after two weeks of daily red wine consumption (375 mL), the maximum concentrations of cunjugated dienes and TBAES in Cu-oxidised LDL were reduced. It was reported that red wine consumption reduced oxidative stress induced by Cu-oxidised LDL and increased HDL cholesterol concentrations. Grape juices showed complete inhibition of copper-induced oxidation of human LDL at the concentration of 0.01% [89]. Phenolic compounds in grapes have showed effective power to regulate the plasma lipid and oxidative stress.

### 4.3. Anticancer Activities

Many evidences have shown that the extracts from grapes and its products had anticancer activity. Hudson *et al*. [90] reported that the grape skin extract induced prostate tumor cell lines apoptosis with high rates. The extract from pomace remaining after wine production inhibited activities of matrix metalloproteinases-2 and -9, and expressed a significant antiproliferative effect on human colon adenocarcinoma cells (Caco-2), which implied by-product of wine would help to fight against carcinogenesis [15,91]. Phenolics of grape juice also significantly inhibited carcinogen-induced DNA adduct formation in rat model [17], and inhibited DNA synthesis in breast cancer cells [16].

Anticancer activities of phenolic compounds from grapes have been studied widely, and the results are summarized in Table 4. Phenolic compounds had dual effects on cells, and modulated cell proliferation was notablely dose-dependent [92]. At high concentration, they were attributed to direct toxic effect and induced cells to death [93].

The relationship between anticancer activity and structure of phenolic compounds was also investigated. The regulation target of grape skin extracts to cell apoptosis was the phosphatidylinositol 3-kinase–Akt and mitogen-activated protein kinase survival pathways. The extracts reduced Akt transcription, and enhanced proteosome degradation [90]. Resveratrol was determined mainly bearing o-diphenoxyl groups, which displayed inhibiting DNA damage induced by ROS, and accelerating DNA damage induced by cupric ions, as well as inducing apoptosis of HL-60 cells, while the composition without such groups did not display the capacity [83].

### 4.4. Anti-inflammation Activities

Phenolic compounds in grapes, especially in grape seeds, have showed significant anti-inflammation effects on rats, mice and human [7,36,102,102], and the contributive molecules may be flavonols, flavanols and procyanidins (oligomeric flavonoids) [7,36,102]. Bralley *et al*. [103] found that extracts from grape skins and seeds inhibited mouse ear inflammation, edema, and polymorphonuclear leukocyte infiltration induced by 12-O-tetradecanoylphorbol 13-acetate, after treated with the extracts for 30 minutes. Moreover, the effect of the combination of grape seeds and skins almost paralleled to that of indomethacin, a common drug against degenerative diseases of joint. These findings indicated that phenolic compounds in grapes possessed obviously anti-inflammatory activity.

The mechanism of anti-inflammation of procyanidins was investigated, and the results showed that it might inhibit releasing proinflammation factors. Immunomodulation was the main pathway, and antioxidative action was another pathway for the anti-inflammation effect of grape phenolics [7,36,104]. Panico *et al*. [36] employed human chondrocytes assays to prove this. After treatment with a combination of extract of grape wine and IL-1b, a notable decrease was detected in the concentration of nitric oxide, prostaglandins E2 and reactive oxygen species in human chondrocytes culture, compared to control groups, and the effects were equal or super to that of indomethacin. Li *et al*. [104] demonstrated that proanthocyanidins could prevent the increase of MDA in rat paws with arthritis induced by carrageenan at the concentration of 10 mg/kg by injection. Nitric oxide synthase activity and *N*-acetyl-β-d- glucosaminidase were also successfully inhibited by proanthocyanidins.

Inhibition or reduction of the cytokine gene expression may be a basic pathway to anti-inflammation for grape phenolics [7,102,104]. After pre-treated with extracts of grape seed procyanidins, human adipocytes and macrophage-like cell lines produced less IL-6 and MCP-1 induced by inflammatory stimulus, and increase in anti-inflammatory adipokine and adiponectin appears. The results demonstrated that grape seeds procyanidins might modulate adipokine and cytokine gene expression related to anti-inflammation [7]. Terra *et al*. [102] reported that grape seed procyanidins inhibited the increase of C-reaction protein in rat plasma induced by high fat feed, and the same trend in IL-6 and TNF-α was detected in the mesenteric white adipose tissue (WAT). Further research demonstrated that CRP mRNA expression was decreased in the liver and mesenteric WAT, while adiponectin mRNA expression was increased in the mesenteric WAT. Then, lipid metabolic disorder and inflammation were availably inhibited. The results indicated that procyanidins in grapes inhibited inflammation at mRNA levels, and major health benefits brought by them involved in decreasing the risk of diseases link to high fat diets and obesity, such as cardiovascular and metabolic disorders.

### 4.5. Antiaging Effects

It was found that polyphenolics presented in foods might be beneficial in reversing the course of neuronal and behavioral aging. Due to their notable antioxidant activity, such as scavenging free radical, they could prevent organs and tissues from oxidative damage, and modify the body negative mechanism of redox status. The evidences were obtained by observing the behaviors of rats, from age 19 to 21 months. After drinking the 10% grape juice, improvements were detected on release of dopamine from striatal slices, as well as cognitive performance in the Morris water maze, while the 50% grape juice improved action capacity [105]. Further research discovered that supplement with grape seed extracts (100 mg/kg b.wt.) for 30 days, phenolic compounds from the extracts inhibited the accumulation of age-related oxidative DNA damages in neural tissue [106]. Balu *et al*. [107] reported the decreased incidence of free radical-induced lipid peroxidation in the central nervous system of aged rats.

### 4.6. Antimicrobial Effects

Plant polyphenols have been demonstrated potential antibacterial [48,107,108], antifungal [28,110] and antiviral [111,112] activities. Rodriguez-Vaquero *et al*. [113] have showed that grape wine inhibited microbial, especially *Escherichia coli* growth, and the inhibition increased as the polyphenol concentration increased, and clarified wines were inactive against all bacteria tested. The extracts of alcohol-free red and white wine exhibited antimicrobial activity to some pathogens such as *Staphylococcus aureus*, *Escherichia coli* and *Candida albicans* [114]. The results suggested that polyphenolic compounds contained in red wines were responsible for the antimicrobial effects. Some studies reported phenolic compounds inhibited other food-borne species such as *Salmonella typhimurium* [115] and *Listerial monocytogenes* [62].

Various bacterial species exhibit different sensitivities towards phenolic compounds. Papadopoulou *et al*. [114] demonstrated *Staphylococcus aureus* were most sensitive to wine extracts, followed by *Escherichia coli* and the least effect of inhibition was detected in *Candida albicans*. The same results were obtained by Radovanovic *et al*. [49], the diameter of the inhibition growth zone for *Staphylococcus aureus* and the zone for *Escherichia coli* were 16–22 mm and 12–20 mm, respectively, and the later exhibited less sensitive to phenolic compounds. Rotava *et al*. [116] showed that phenolic compounds from defatted grape (*Vitis vinifera*) seed extract inhibited the growth of *Staphylococcus aureus* and *Escherichia coli*, while they showed no effects on *Salmonella* sp. Rodriguez-Vaquero *et al*. [21] showed that *Flavobacterium* sp. was not inhibited by all any phenolic compounds tested. The time of reaction were also different, for example, Karapinar *et al*. [117] demonstrated that koruk (unripe grape from *Vitis vinifera*) juice immediately decreased the initial populations of *Salmonella typhimurium* at 1–3.5 log cfu/g. But for some microbial species, the antibacterial activity acted too slowly. Baydar *et al*. [109] showed that grape seed extract acted against *Staphylococcus aureus* after 48 hours and *Aeromonas hydrophila* after one hour.

The phenolic compounds from different parts of grapes displayed different antimicrobial effects. The antimicrobial activity of fermented pomace was either as effective as or significantly better than whole fruit grape extracts [118]. Some researches showed that seed extracts were more effectively antimicrobial than other parts of grapes. The experimental study showed the minimum inhibition concentration (MICs) of seed and stem extracts for antilisterial were 0.26 and 0.34 mg GAE/L, respectively [119]. The extracts from whole grape fruit inhibited bacterial growth at concentrations of 680 mg GAE/L and 1360 mg GAE/L for Gram(+) and Gram(−) bacteria, respectively. Jayaprakasha *et al*. [109] showed grape seed extracts inhibited bacterial growth at 340–390 mg GAE/L and 475–575 mg GAE/L for Gram(+) and Gram(−) bacteria, respectively. The extract of grape leaves also exhibited less antimicrobial activity than seed extracts. The extract from grape flesh did not exhibit any antimicrobial effect at all [120]. Brown *et al*. [121] showed that the grape skin possessed the strongest activity in anti-*Helicobacter pylori*, followed by grape synergy (skin and seed) and seed. The increase order of the antimicrobial activity was flesh, whole fruit grape extracts, fermented pomace, skin, leave and seed.

Phenolic compounds in grape such as resveratrol displayed potent antifungal activity against the human pathogenic fungi *Candida albicans* at concentrations of 10–20 μL. The notable benefit of phenolics was no induction of hemolytic activity against human erythrocytes, compared to chemical medicines [110]. Anastasiadi *et al*. [119] suggested that high concentration of flavonoids and their derivatives in grape seeds and flavonoids, stilbenes, and phenolic acids in grape stem were responsible for the antimicrobial activity. Rodriguez–Vaquero *et al*. [20] concluded that the non-flavonoid caffeic acid and the flavonoids rutin and quercetin were the compounds with higher inhibitory activities on *Listerial monocytogenes* growth. Rhodes *et al*. [21] showed that polymeric phenolic fractions acted the highest inhibition activity for all *Listerial* species, but not for other bacteria, such as *Bacillus cereus*, *Salmonella Menston*, *Escherichia coli*, *Staphylococcus aureus* or *Yersinia enterocolitica*. The red-pigmented polymeric phenolics from juice and skin showed pH-dependent antilisterial activity, while the unpigmented polymeric phenolics from the seed showed antilisterial activity which was independent of pH, as some phenolic acids acted.

The relationship between compound structure and antimicrobial activity has been investigated. The core structures with 3,4,5-trihydroxyphenyl groups found in epigallocatechin, epigallocatechin-3-O-gallate, castalagin and prodelphinidin might be important for antibacterial activity. This indicated that the number of hydroxyls and the degree of polymerizzation might be pivotal for antimicrobial activity of phenolic compounds [122]. According to anti-rabies activity of 24 phenolic compounds, Chavez *et al*. [112] considered that free hydroxyl and ether groups mainly influenced the anti-rabies activity. Employing herpes simplex virus (HSV) and human immunodeficiency virus (HIV), De Bruyne *et al*. [111] found epicatechin-containing dimer and the presence of *ortho*-trihydroxyl groups in the B-ring were important for anti-HSV, radical-scavenging and immunological activities. Thtmothe *et al*. [118] demonstrated that the different concentration of anthocyanins and flavonols notablely decreased the activity of glucosyltransferases B and C (70%–85%) in *Streptococcus mutans* cells at total concentrations 62.5 μg/mL. At the same time, F-ATPase activity was reduced 30–65% at 125 μg/mL. The result suggested that conjugation of phenolic and protein in microorganism, especially key enzyme might be major pathway to inhibit the growth of microorganism.

The application of phenolic compounds could be better in food preservation than in medical field [54,62], and the potent function of phenolics as perfect nature preservative and antimicrobial agents for food is very promising. In Turkish diet, koruk juice is used as flavoring and acidifying agent. It has acted as a practicable antimicrobial agent for salad vegetables unconsciously due to its immediate inhibition against *Salmonella typhimurium* [117]. In order to check the effect of protection food from microbial infecting, Sivarooban *et al*. [115] exposed several species of microorganism to the soy protein isolate film with GSE 1%, nisin 10,000 IU/g, and EDTA 0.16%. This film reduced *Listerial monocytogenes* populations by 2.9 log CFU/mL, and *Escherichia coli* O157:H7 and *Salmonella typhimurium* were reduced by 1.8 and 0.6 log CFU/mL, respectively. This finding suggested the potential applications of phenolic compounds to maintain shelf life, and improve safety of ready-to-eat food products.

The antioxidant, cardioprotective, anticancer, anti-inflammation, antiaging and antimicrobial activities of grapes and its products have been discussed above. Finally, the bioactivities of phenolic compounds from grapes are summarized in Table 5. As shown in Table 5, the phenolic compounds have a variety of bioactivities.

## 5. Bioavailability

Several studies showed rapid absorption of the polyphenolics, such as procyanidins, quercetin and flavanols from grapes into plasma, with plasma concentrations peaking at two or three hours after ingestion [31,48,87,127–129]. The increase of lipid-bound polyphenolics in serum could be detected, and as a result of the bioactivity of polyphenolics, significant decrease was detected on lipid peroxidation in serum [48]. Moreover, after two weeks of daily red wine consumption (375 mL), plasma levels of total phenolic concentrations increased significantly, and trace levels of metabolites, mainly glucuronides and methyl glucuronides of (+)-catechin and (−)-epicatechin, were detected in plasma, which could not be found in a control group [14]. These results indicated that phenolic compounds could be absorbed by human digestion system, and entered the blood successfully. The phenolic compounds in the extracts from defatted mill grape seed acted bioactive function by protecting the isolated rat hepatocytes from oxidative stress induced by anticancer drugs. In order to research the mechanisms involved in pathways of phenolic compounds entering into cell, Laurent *et al*. [33] employed an *in vitro* digestion/Caco-2 cell culture model. However, no phenolic compounds were detected in the basal compartment of transwells or in cell monolayers. They also showed that the availability of phenolic compounds was not affected by salivary and gastric incubations but decreased during intestinal digestion.

The mechanisms involved in the process of digestion and absorption of phenolic compounds in gastrointestinal lumen are complex and not very clear. Some results showed that phenolic compounds were able to chelate to iron. Presence of iron and phenolic compounds has been found in the lumen during digestion, during which iron–polyphenol interacted and formed iron-chelating complexes [22], which provoked a more marked decrease in the concentration of hydroxycinnamic derivatives, flavones and flavan-3-ols compared to the control assays during *in vitro* gastointestinal digestion. *In vitro* digestion, Argyri *et al*. [130] demonstrated that red wine decreased the concentration of digest phenolics attributable to the formation of iron-polyphenolic chelates. By molecule analyzing, the interaction of iron and polyphenolic involved the chemical structures of hydroxyl groups, as reported in flavonoids: ortho-dihydroxyl groups, the presence of 5-OH and/or 3-OH in conjunction with a C4 keto group, and a large number of OH groups [131].

It was found that phenolic compounds have affinities with some proteins after absorbed [24,25,132,133]. Employing *in vitro* digestion/ Caco-2 cell culture model, Laurent *et al*. [33] found about 43.9% of catechin, 85.3% of epicatechin and all dimers disappeared at the end of 2 h of intestinal incubation, associating with a decrease of some cells enzyme activities, such as alkaline phosphatase and sucrase-isomaltase aminopeptidase N. The results showed that phenolics had interacted with pancreatic proteins, which were detected by unmasked by acetonitrile extraction. Polyphenols also seemed to have affinities with enterocyte brush border enzymes [132]. Some researchers showed that phenolic compounds had strong affinities with proteins and particularly with human salivary prolinerich proteins and histatins [24,25,133] to form both non-covalent and covalent associations according to the phenolic compound size. Flavonoids were strongly affected by the presence of milk, especially after the digestion process [129]. Procyanidins from grape seed extracts strongly combined to milk protein attributing to the higher degree of polymerization. The insoluble complexes, such as protein-tannins, were stable throughout the digestive tract [131,133]. However, the fate of the complexes of low molecule weight phenolics and protein is still unclear.

Decomposition of phenolic compounds caused by pH changes has been shown in digestion lumen. After two hours of *in vitro* incubation, monomers and dimers were quite stable at pH 7 in intestine medium, but 20% dimers were degraded at pH 7.4, and all dimers disappeared in pH 8.5. 15%–34% of epicatechin were degraded at pH 7.5 with incubation for two hours, while catechin was stable [134]. At pH 2, decomposition of high polymerized oligomers (>trimers) of procyanidins might occur and the slight increase in dimers procyanidins was observed through gastric step [135]. Flavanols and flavonols monomers and dimers were stable at acidic condition [136,137]. Anthocyanins could be digested completely in the contents of large intestine of freshly slaughtered pigs after six-hour incubation, and the metabolites were mainly 3–*O*-methylgallic acid, syringic acid and 2,4,6-trihydroxybenzaldehyde [138].

## 6. Potential Toxicity

The potential toxicity of some polyphenols from grape, such as epicatechin to the fibroblast, and keratinocyte cell lines, has been investigated. After exposing the two cell lines to epicatechin for 24 hours or more time, the notablely negative effects were observed when the concentration was 3–7 fold higher than that of expressing positively antioxidant activity. Moreover, the compounds with a gallate group exhibited more potential toxicity than those without the gallate group [23]. In addition, noticeable DNA damage was induced in mice spleen cells by incubating with higher concentration (150 μmol/L) of catechin [98]. Grape extracts was also found to promote mitomycin C inducing sister chromatid exchange at concentration from 75 to 300 μg/mL in human peripheral blood lymphocytes [139]. The compounds with polyphenols, caffeic acid, gallic acid, and rutin hydrate enhanced MMC-induced clastogenicity at accordant concentrations. The results suggested that negative effects of phenolic compounds were related to the synergistic effect of some molecules, and the concentration was not always a crucial factor. Therefore, the dose and composition of grape extracts should be investigated further for secure and healthy application of grape products.

## 7. Conclusions and Future Prospects

Grape and products from grape have been consumed for a long time. The studies have demonstrated an inverse association between intake of grape and products from grape and mortality from age-related diseases such as coronary heart diseases. The health benefits of grapes are thought to arise mainly from bioactivities of their polyphenols. Anthocyanins, flavonoids and resveratrol are the major functional components that are responsible for most of biological activities of grape. Tremendous progress has been obtained for the extraction, analysis and biological activities of polyphenols in grape. The bioactive compounds were usually extracted from grape using the liquid-liquid extraction, and high-performance liquid chromatography with UV or MS detection could be applied to analysis of active components in grape. The grape and its main components anthocyanins, flavonoids and resveratrol have a variety of bioactivities, such as antioxidant, cardioprotective, anticancer, anti-inflammation, antiaging and antimicrobial activities, which are closely related to the prevention against disease and promotion of health, making greater potential for grape in the field of food and pharmaceutical application. The structure-activity relationships of some polyphenols have been studied, and the results obtained could be used to modify structure of polyphenol as well as to design and synthesize novel polyphenols with special function. Most of phenolic compounds were bioavailable, but some high molecular weight phenolics could not be absorbed. In addition, the effect of some phenolic compounds was negative on health at higher concentration, and some structures promoted the negative effect.

In the future, the extraction methods of polyphenols from grape should be improved, and the by-products of wine industry should be utilized effectively. The crude extracts from grape could be used as diet supplements for health-protection after defining the levels or limits to make sure the dose is safe for health, but bioactive components at high purity should be used instead of crude extracts in medicinal preparations from grape. In order to explore more effective functional food or pharmaceutical products based on grape, more wide pharmacological studies should be carried out to determine new pharmacodynamic effects, such as anti-influenza, anti-obesity and antidiabetic activities. The relationship of structure-activity should be studied further, and the key mechanisms of bioactivities should be understood clearly. In addition, more attention should be paid to minor components in grape because special pharmacodynamic effects could be found from minor components. The structural diversities and pronounced biological activities of compounds in grape indicate that grape are worthy of further studies that may lead to the identification of new functional constituents. The polyphenols from grape will widely be employed to prevent and treat these diseases in association with reactive oxygen species, such as atherosclerosis, coronary heart diseases and cancer.

## Figures and Tables

**Table 1.** The phenolic compounds in different parts of grape and its products.

**Resource**	**Phenolic compounds**	**References**
seed	gallic acid, (+)-catechin, epicatechin, dimeric procyanidin, proanthocyanidins	[26,30–32]
skin	Proanthocyanidins, ellagic acid, myricetin, quercetin, kaempferol, trans-resveratrol	[26,30]
leaf	myricetin, ellagic acid, kaempferol, quercetin, gallic acid	[26]
stem	rutin, quercetin 3-O-glucuronide, trans-resveratrol, astilbin	[27]
raisin	hydroxycinnamic acid, hydroxymethylfurfural	[34]
red wine	malvidin-3-glucoside, peonidin-3-glucoside, cyanidin-3-glucoside, petunidin-3-glucoside, catechin, quercetin, resveratrol, hydroxycinnamic acid	[35–37]

**Table 2.** The antioxidant capacities of the extracts from different parts of grape and its products.

**Resource**	**TEAC**^a^	**FRAP**	**DPPH**	**ORAC**	**Ref.**
grape pomace	0.91 g/L (EC_50_)	-	0.20 g/L (EC_50_)	-	[51]
grape seed	-	-	>663 μmol TE/g	-	[62]
defatted grape seed	36.36 mol TE/100 g	21.6 mol TE/100 g	-	-	[47]
whole seed	76.3 mol TE/100 g	58.04 mol TE/100 g	-	-	
grape seed	-	-	16.8 to 92 mmol TE/g	42.18 mmol TE/g	[63]
grape skin	-	-	15.7 to 113.3 mmol TE/g	36.40 mmol TE/g	
grape seed	281.3 μmol TE/g	-	-	-	[26]
grape leaf	236.1 μmol TE/g	-	-	-	
grape skin	12.8 μmol TE/g	-	-	-	
grape flesh	2.4 μmol TE/g	-	-	-	
grape juice	25 mmol TE/L	32 mmol Fe^2+^/L	15 mmol TE/L	-	[48]
grape wine	-	8.8 μmol TE/g	22.9 to 26.7 μmol TE/g	-	[64]
grape wine	-	3.098 mg TE/L	70.7% inhibition	10.724 μmol/L	[65]

a TE is Trolox^®^ antioxidant equivalent.

**Table 3.** Antioxidant activities of the extracts from grapes and its products.

**Resource**	**Antioxidant activity**	**References**
grape seed	decreasing the oxidated LDL in plasma	[69]
juice	reducing oxidative stress in serum	[48]
red wine	protection against membrane oxidation of *Saccharomyces cerevisiae* induced by H_2_O_2_	[70]
fruit beverage (grape+orange+apricot)	protecting mitochondrial and the antioxidant system against oxidative stress induced by H_2_O_2_	[71]
grape wine	protecting hypercholesterolemic hamsters against aortic fatty streak accumulation	[37]
defatted milled grape seed	dealing with the oxidant stress induced by chemical anticancer adriamycin; reducing TBAS and elevating the levels of GSH and ATP	[72]
grape seed extract	food preservatives for fish flesh and oil	[62]
white grape dietary fiber concentrate	antioxidation for polyunsaturated fatty acid in oil	[73]

**Table 4.** Anticancer activities of phenolic compounds from grapes.

**Phenols**	**Subject**	**Effects**	**References**
proanthocyanidins	mouse mammary carcinoma cell line	inhibited breast cancer metastasis	[94]
anthocyanin	rat liver clone 9 cells	activated antioxidant response element upstream of genes	[95]
	colon cancer cell lines (HT-29 and Caco-2)	induced 2–4 times increase in DNA fragmentation	[96]
	vascular tumor biology	repaired and protected genomic DNA integrity and retard blood vessel growth in some tumors	[97]
procyanidin, catechin or gallic acid	mice spleen cells	inhibited DNA damage induced by hydrogen peroxide	[98]
catechin	human breast cancer cell line	decreased cell viability and proliferation at 30 and 60 μg/mL	[74]
procyanidins		decreased cell viability and proliferation at 30, but not 60 μg/mL	
flavone	human colon carcinoma HT-29 cells	reduced cell proliferation with an EC50 value of 54.8 ± 1.3 μmol/L, induced differentiation and apoptosis	[99]
flavonoid	HT-29 cells	more effectively induced apoptosis than antitumor agent camptothecin	
resveratrol	prostate cancer cell lines	induced apoptotic and antiproliferative effects at ≥ 15 μmol/L and above 24 hours	[100]
	human mammary epithelial cells	inhibited cyclooxygenase-2 transcription	[101]

**Table 5.** Bioactivities of some phenolic compounds from grapes.

**Phenolic compound**	**Bioactivity**	**References**
resveratrol	free radical scavenging	[76,81]
	antiproliferation	[83,100]
	enhancing plasma NO level	[123]
	regulating lipid metabolism	[37]
	protection against membrane oxidation	[124]
quercetin	antibacterial	[20]
	enhancing plasma NO level	[123]
catechin	anticancer	[74]
	free radical scavenging	[13,68,83]
	antibacterial	[119]
	anti-inflammation	[36]
	protection against membrane oxidation	[124]
flavone	antiproliferation	[99]
flavonol	free radical scavenging	[75,81]
procyanidin	anticancer	[74,94]
	free radical scavenging	[75]
	anti-inflammation	[8,102,125]
	antioxidant	[89]
anthocyanin	vasorelaxation	[79]
	free radical scavenger	[97]
	antibacterial	[118,119]
	antioxidant	[89]
	inducing apoptosis	[126]
gallic acid	free radical scavenger	[76]
epicatechin	antibacterial	[76,118]
